# A multicenter analysis of genomic profiles and PD-L1 expression of primary lymphoepithelioma-like carcinoma of the lung

**DOI:** 10.1038/s41379-019-0391-9

**Published:** 2019-10-28

**Authors:** Zhanhong Xie, Laiyu Liu, Xinqing Lin, Xiaohong Xie, Yingying Gu, Ming Liu, Jiexia Zhang, Ming Ouyang, Analyn Lizaso, Hua Zhang, Weineng Feng, Bing Li, Han Han-Zhang, Shuyin Chen, Shiyue Li, Nanshan Zhong, Hao Liu, Chengzhi Zhou, Yinyin Qin

**Affiliations:** 1 State Key Laboratory of Respiratory Disease, National Clinical Research Center for Respiratory Disease, Guangzhou Institute of Respiratory Health, The First Affiliated Hospital of Guangzhou Medical University, Guangzhou, 510120 Guangdong China; 2 Chronic Airway Diseases Laboratory, Department of Respiratory and Critical Care Medicine, Nanfang Hospital, Southern Medical University, Guangzhou, 510515 Guangdong China; 3 Burning Rock Biotech, Guangzhou, 510300 Guangdong China; 4 Department of Head and Neck/Thoracic Oncology, The First People’s Hospital of Foshan, Foshan, 528000 Guangdong China

**Keywords:** Lung cancer, Cancer genetics

## Abstract

To understand the molecular mechanism of tumorigenesis of pulmonary lymphoepithelioma-like carcinoma and explore potential therapeutic strategies, we investigated the genomic profiles and PD-L1 expression of 29 Chinese pulmonary lymphoepithelioma-like carcinoma patients at various stages. We performed capture-based targeted sequencing on tissue samples collected from 27 patients with sufficient samples using a panel consisting of 520 cancer-related genes, spanning 1.64 Mb of the human genome. We identified 184 somatic mutations in 109 genes from 26 patients. One patient had no mutations detected by this panel. Copy number variations were detected in 52% (14/27) of the patients, with a majority having advanced-stage disease (10/14). Except for the detection of *ERBB2* amplification and *KRAS* mutation in two patients, no other classic lung cancer driver mutations were detected. Interestingly, 78% (21/27) of the patients had mutations in epigenetic regulators. Of the 184 mutations identified, 51 occurred in 29 epigenetics-related genes. Furthermore, we performed PD-L1 immunohistochemistry staining using the Dako 22C3 assay and demonstrated that 69% (20/29) of the cohort had positive PD-L1 expression, of which three patients received and benefited from a PD-1 inhibitor. In conclusion, we elucidated a distinct genomic landscape associated with pulmonary lymphoepithelioma-like carcinoma with no classic lung cancer driver mutation but an enrichment of mutations in epigenetic regulators. The detection of high PD-L1 expression and lack of any canonical druggable driver mutations raises the potential of checkpoint immunotherapy for pulmonary lymphoepithelioma-like carcinoma.

## Introduction

A majority of lymphoepithelial carcinoma originates from the nasopharynx, but some will involve the lung, salivary glands, stomach, urinary tract, and rarely ovaries [1, 2]. Pulmonary lymphoepithelioma-like carcinoma is a rare and distinct subtype of non-small-cell lung cancer with an incidence of ~0.7% of all non-small cell lung cancer cases. Pulmonary lymphoepithelioma-like carcinoma tends to affect young, non-smoking patients of Asian ethnicity [3–9]. It was previously classified as a variant of large cell carcinoma [10] but has recently been reclassified under other and unclassified carcinomas in the 2015 World Health Organization (WHO) classification of lung tumors [11]. Its histology was first described as an Epstein-Barr virus-associated epithelial neoplasm in 1987 [12] and resembles undifferentiated nasopharyngeal carcinoma with predominant lymphocytic infiltration [3, 11, 13]. Interestingly, non-classic histologic morphologies have been observed for pulmonary lymphoepithelioma-like carcinoma including EBV negative [14] and heterogeneity in the extent of lymphocytic infiltration [15]. Nonetheless, the circulating serum EBV DNA can serve as a potential marker for disease and treatment monitoring, particularly among Asian pulmonary lymphoepithelioma-like carcinoma patients [3–8, 14, 16–18].

Over the past 32 years since its discovery, ~500 cases have been reported worldwide. Due to its rarity, no clinical trials have been conducted to establish standard treatment. Since most patients are diagnosed at an early stage, the prognosis for pulmonary lymphoepithelioma-like carcinoma patients is more favorable compared with other types of lung cancer, with a median overall survival of about 107 months vs. 13 months for non-pulmonary lymphoepithelioma-like carcinoma patients and a 5-year survival of about 60% [5, 7]. The main treatment strategy for early-stage disease is surgery [3, 6, 7]. Unresectable patients often undergo multimodal therapy, primarily consisting of chemotherapy and radiotherapy with better prognosis than other types of non-small-cell lung cancer [19]. Despite the efficacy of multimodal treatment for advanced-stage disease [19–22], chemotherapy and radiation regimen has not reached consensus for this subset of patients. In addition, the use of targeted therapy and immunotherapy in pulmonary lymphoepithelioma-like carcinoma is limited due to the lack of information regarding the molecular mechanism of its tumorigenesis. In efforts to understand the molecular pathways involved in the development of pulmonary lymphoepithelioma-like carcinoma, a number of studies have examined the mutation status of classic lung cancer oncogenic driver and tumor suppressor genes, including *EGFR*, *KRAS*, *BRAF*, *ALK*, *ROS1*, and *TP53* [4]; however, all of these common oncogenic drivers were often not mutated, indicating the involvement of other pathways in its tumorigenesis [4, 6, 7, 15, 23, 24]. In order to develop novel therapeutic strategies for pulmonary lymphoepithelioma-like carcinoma patients, their mutation landscape needs to be elucidated to shed light on the potential mechanisms of its tumorigenesis and to discover drug targets. In this study, we determined the mutation profile and the expression of programmed death-ligand 1 (PD-L1) of 29 Chinese pulmonary lymphoepithelioma-like carcinoma patients at various disease stages.

## Patients and methods

### Patients

Twenty-nine Chinese patients diagnosed with pulmonary lymphoepithelioma-like carcinoma in the three participating hospitals from Guangdong Province (The First Affiliated Hospital of Guangzhou Medical University, Nanfang Hospital and The First People's Hospital of Foshan), between July 2015 and December 2018 were recruited for this study. Pulmonary lymphoepithelioma-like carcinoma were diagnosed according to the criteria by the 2015 WHO histological classification of lung tumors [11]. All the tumors were evaluated by two independent pathologists. Pathologic or clinical staging was according to the seventh edition of the American Joint Committee on Cancer [25]. Tumor assessment for treatment response was investigator-assessed based on Response Evaluation Criteria in Solid Tumors version 1.1 [26]. Medical records were retrieved to collect clinicopathologic data, treatment history, and survival outcome. This study has been approved by the relevant Institutional Review Board of all the participating hospitals (Approval number: ChiCTR-DDD-16008065). Written informed consent was provided by all the patients included in the study.

### Tissue DNA isolation and capture-based targeted DNA sequencing

Tissue DNA was extracted from formalin-fixed, paraffin-embedded tumor tissues using QIAamp DNA formalin-fixed paraffin-embedded tissue kit (Qiagen, Hilden, Germany). A minimum of 50 ng of DNA is required for NGS library construction. Tissue DNA was sheared using Covaris M220 (Covaris, MA, USA), followed by end repair, phosphorylation, and adapter ligation. Fragments between 200–400 bp from the sheared tissue DNA were purified (Agencourt AMPure XP Kit, Beckman Coulter, CA, USA), followed by hybridization with capture probes baits, hybrid selection with magnetic beads, and PCR amplification. The quality and the size of the fragments were assessed using the Qubit 2.0 fluorometer with the dsDNA high-sensitivity assay kit (Life Technologies, Carlsbad, CA). Indexed samples were sequenced on Nextseq500 (Illumina, Inc., USA) with paired-end reads and average sequencing depth of 1000× using a panel with 520 cancer-related genes, spanning 1.64 megabases (Mb) of the human genome (OncoScreen Plus, Burning Rock Biotech, Guangzhou, China). The genes included in the panel are listed in Table S1.

### Sequence data analysis

Sequence data were mapped to the reference human genome (hg19) using the Burrows–Wheeler Aligner v.0.7.10 [27]. Local alignment optimization, duplication marking, and variant calling were performed using the Genome Analysis Tool Kit v.3.2 [28], and VarScan v.2.4.3 [29]. Tissue samples were compared against their own white blood cell control to identify somatic variants. Variants were filtered using the VarScan fpfilter pipeline, loci with depth <100 were filtered out. Base calling in plasma and tissue samples required at least eight supporting reads for single nucleotide variations and two and five supporting reads for insertion-deletion variations, respectively. Variants with population frequency over 0.1% in the ExAC, 1000 Genomes, dbSNP or ESP6500SI-V2 databases were grouped as single nucleotide polymorphisms and excluded from further analysis. Remaining variants were annotated with ANNOVAR (2016-02-01 release) [30] and SnpEff v.3.6 [31]. Analysis of DNA translocation was performed using Factera v.1.4.3 [32]. Copy number variations were analyzed based on the depth of coverage data of capture intervals. Coverage data were corrected against sequencing bias resulting from the GC content and probe design. The average coverage of all captured regions was used to normalize the coverage of different samples to comparable scales. Copy number was calculated based on the ratio between the depth of coverage in tumor samples and average coverage of an adequate number (*n* > 50) of samples without copy number variations as references per capture interval. Copy number variation is called if the coverage data of the gene region was quantitatively and statistically significant from its reference control. The limit of detection for copy number variations is 1.5 and 2.64 for deletions and amplifications, respectively.

Tumor mutation burden per patient was computed as a ratio between the total number of nonsynonymous mutations detected with the total coding region size of the panel used using the formula below. Since copy number variations, fusions, large genomic rearrangements, and mutations occurring on the kinase domain of *EGFR* and *ALK* were excluded from the mutation count, the total size of the coding region of the panel for estimating tumor mutation burden is 1.26 Mb for the 520-gene OncoScreen Plus panel.

$$	{\!}{\mathrm{Tumor}}\;{\mathrm{mutation}}\;{\mathrm{burden}} \\ {\!\!}	= \frac{{{\mathrm{mutation}}\;{\mathrm{count}}({\mathrm{except}}\;{\mathrm{for}}\;{\mathrm{copy}}\;{\mathrm{number}}\;{\mathrm{variations}}\;{\mathrm{and}}\;{\mathrm{fusion}})}}{{{\mathrm{total}}\;{\mathrm{size}}\;{\mathrm{of}}\;{\mathrm{coding}}\;{\mathrm{region}}\;{\mathrm{of}}\;{\mathrm{the}}\;{\mathrm{panel}}\;{\mathrm{used}}}}.$$

### Immunohistochemical staining of PD-L1

Formalin-fixed paraffin-embedded tumor samples were stained with PD-L1 antibody (clone 22C3) on the Dako automated staining platform following the manufacturer’s standard protocol (Dako 22C3 PharmDx Assay, Dako Autostainer Link 48, Agilent, Santa Clara, CA, USA). All the areas in each tissue section were evaluated for the PD-L1 expression. Tissue sections evaluated to have moderate to strong membrane staining in at least 5% of the tumor cells are considered to be positive for PD-L1 overexpression, while tissue sections with an absence or detection of staining in less than 5% of the cells were considered to be negative [33, 34]. PD-L1 expression was expressed as tumor proportion score, with scores of 0–5% as negative, between 5 and 49% as low expression and ≥50% as high expression level. All the slides were scored for PD-L1 membrane staining by two independent pathologists.

### Statistical analysis

All the data were analyzed using R statistics package (R version 3.4.0; R: The R-Project for Statistical Computing, Vienna, Austria). Differences in the groups were calculated and presented using Fisher’s exact test, paired, two-tailed Student’s *t* test or analysis of variance, as appropriate. *P* value with *P* < 0.05 was considered as statistically significant.

## Results

### Histopathologic features of pulmonary lymphoepithelioma-like carcinoma

Lymphoepithelioma is defined by the presence of clusters of epithelial tumor cells with round, oval, or elongated nuclei, surrounded by lymphoid cells including lymphocytes and granulocytes (Fig. S1A). Immunohistochemical staining revealed positive immunoreactivity for cytokeratin 5/6 and P63 in all the pulmonary lymphoepithelioma-like carcinoma tumors in the cohort (Fig. S1B and S1C). In addition, in situ hybridization of EBV-encoded small non-polyadenylated RNA (EBER) also revealed positive EBER transcription in all the patient samples (Fig. S1D).

### Patient characteristics

The cohort had 52% (15/29) females and 48% (14/29) males. The median age was 55, ranging from 24 to 76 years. Less than half of the cohort (38%, 11/29) were smokers and one patient had regular exposure to second-hand smoke, while 59% (17/29) were non-smokers. The clinical characteristics of the patients were summarized in Table 1.

**Table 1:** Clinicopathologic characteristics of the cohort

Clinicopathologic characteristics			*n* (%) *n* = 29
Gender	Male		14 (48.3%)
	Female		15 (51.7%)
Age (years) (median, range)			55 (24–76)
Smoking history	Smoker or with smoking history		12 (41%)
	Non-smoker		17 (59%)
Disease stage	Early-stage	IA	0
		IB	1 (3.4%)
		IIA	2 (6.9%)
		IIB	3 (10.3%)
		IIIA	7 (24.1%)
	Advanced-stage	IIIB	4 (13.8%)
		IIIC	2 (7.4%)
		IVA	6 (20.7%)
		IVB	4 (13.8%)
Surgery	Yes		10 (34.5%)
	No		19 (65.5%)
Radiotherapy	Yes		5 (17%)
	No		24 (83%)
Local or systemic regimen received	Neoadjuvant + surgery + adjuvant chemotherapy		4 (14%)
	Surgery + adjuvant chemotherapy		5 (17%)
	Chemotherapy alone		13 (48%)
	Chemotherapy + radiotherapy		3 (10%)
	Surgery alone		1 (3%)
	Radiotherapy alone		1 (3%)
	No local or systemic regimen received		2 (7%)
Best response	Stable disease		12 (44%)
	Partial response		7 (26%)
	Unknown		8 (30%)
Survival status	Still alive		29 (100%)
Follow-up (months) (median, range)			14.2 (0.2–41.4)
PD-L1 status	High expression (TPS ≥ 50%)		4 (14%)
	Low expression (TPS 5–49%)		16 (55%)
	Negative		9 (31%)

Of the 29 patients in the cohort, 45% (13/29) were diagnosed with early-stage (Stage IA to IIIA) and 55% (16/29) with advanced-stage (Stage IIIB to IVB) disease, with 1 stage IB, 2 stage IIA, 3 stage IIB, 7 stage IIIA, 5 stage IIIB, 2 stage IIIC, 5 stage IVA, and 4 stage IVB. For patients with early-stage disease, 77% (10/13) underwent surgery, including one with radical resection and nine received adjuvant and/or neoadjuvant chemotherapy. One stage IIIA patient also received postoperative radiotherapy. The remaining three patients were treated with chemotherapy of gemcitabine combined with either nedaplatin or carboplatin as the first-line treatment. For patients with advanced disease, except for the two patients who refused to receive treatment, all the patients received chemotherapy. Of the 14 chemotherapy-treated patients, two also had concurrent radiotherapy. Among the ten patients who had records for the best response, five achieved partial response and five achieved stable disease. All 29 patients are still alive with a median follow-up of 14.2 months, as of the last follow-up on December 28, 2018. The detailed clinical features of each of the 13 early-stage and 16 advanced-stage patients in the cohort were listed in Tables 2 and 3, respectively.

**Table 2:** Clinical features of the 13 early-stage pulmonary lymphoepithelioma-like carcinoma patients

Patient ID	Age	Gender	Smoking status, smoking pack years	Disease stage	Treatment regimen received	Best response	Immunotherapy, best response	Number of CNVs detected	TMB (mutations/Mb)	PD-L1 TPS (%)	Degree of lymphoid cell infiltration	Date of diagnosis	OS as of last follow-up date (months)	Survival status
P05	59	Male	Smoker, 20	IB	RT	SD	No	0	3.2	0	10%	2017/2/8	14.0	Alive
P17	38	Male	Smoker, 40 (10 years water pipe)	IIA	S + adj CT	SD	No	0	1.6	20	40%	2017/1/4	19.9	Alive
P22	55	Male	Smoker, 40	IIA	S + adj CT	–	No	0	0.8	5	40%	2018/12/1	0.1	Alive
P04	39	Male	Smoker, 10	IIB	S + adj CT	SD	No	0	0^a^	0	50%	2016/12/7	14.9	Alive
P08	56	Female	No	IIB	Neo CT + S + adj CT	–	Nivolumab, SD	7	27.8	30	20%	2017/10/2	14.2	Alive
P24	57	Female	No	IIB	S	–	No	0	2.4	60	10%	2018/12/13	0.2	Alive
P01	43	Male	Smoker, 1	IIIA	Neo CT + S + adj CT + RT	SD	No	0	1.6	0	40%	2015/7/1	41.4	Alive
P02	55	Female	No	IIIA	Neo CT + S + adj CT	SD	No	0	1.6	30	40%	2016/10/31	6.3	Alive
P23	48	Male	Smoker, 15	IIIA	S + adj CT	SD	No	0	1.6	2	40%	2015/7/26	39.9	Alive
P14	58	Female	No	IIIA	S + adj CT	SD	No	2	3.2	2	40%	2015/8/7	37.2	Alive
P20	64	Male	Smoker, 10	IIIA	CT	PR	No	7	4	0	30%	2018/4/17	7.5	Alive
P27	64	Female	No	IIIA	CT	–	No	0	3.2	20	50%	2018/11/23	0.9	Alive
P15	47	Female	No	IIIA	CT	PR	No	2	0.8	50	5%	2016/9/15	27.4	Alive

– Data not available

*Adj CT* adjuvant chemotherapy, *CNV* copy number variation, *CT* chemotherapy, *Neo CT* neoadjuvant chemotherapy, *OS* overall survival, *PR* partial response, *RF* relapse-free, *RT* radiotherapy, *S* surgery, *SD* stable disease, *TMB* tumor mutation burden, *TPS* tumor positive score

^a^Patient P04 was wild-type in all the genes in the panel used

**Table 3:** Clinical features of the 16 advanced-stage pulmonary lymphoepithelioma-like carcinoma patients

Patient ID	Age	Gender	Smoking status, smoking pack year	Disease stage	Treatment regimen received	Best response	Immunotherapy, best response	# of CNVs detected	TMB (mutations/Mb)	PD-L1 TPS (%)	Degree of lymphoid cell infiltration	Date of diagnosis	OS as of last follow-up date (months)	Survival status
P09	48	Male	No	IIIB	CT	PR	No	2	0	0	40%	2016/6/27	30.0	Alive
P19	55	Female	No	IIIB	CT	–	No	0	1.6	10	40%	2018/11/13	1.0	Alive
P21	66	Female	No	IIIB	CT	SD	No	0	0.8	0	1%	2018/8/21	3.7	Alive
P28^a^	49	Female	No	IIIB	CT		Nivolumab, SD		–	60	5%	2017/4/12	19.0	Alive
P25	45	Male	Smoker, 3	IIIC	CT	–	No	7	6.3	0	30%	2018/5/5	6.7	Alive
P26	57	Male	Smoker, 40	IIIC	CT + RT	SD	No	1	2.4	10	30%	2018/6/10	4.6	Alive
P06	32	Female	Non-smoker (exposure to second-hand smoke)	IVA	Neo CT + S + adj CT	PR	No	7	2.4	10	8%	2016/11/17	25.3	Alive
P07	76	Male	No	IVA	CT + RT	SD	No	8	3.2	0	10%	2017/4/6	20.7	Alive
P13	49	Male	Smoker, 20	IVA	CT	PR	No	1	0	30	30%	2018/7/13	5.0	Alive
P12	60	Female	No	IVA	CT	PR	No	4	15.9	30	10%	2018/7/9	4.9	Alive
P03	65	Female	No	IVA	Not treated	–	No	5	0.8	10	30%	2016/11/28	22.7	Alive
P29^a^	48	Male	No	IVA	CT		SHR-1201, SD	–	–	15	5%	2017/2/28	13.0	Alive
P11	24	Female	No	IVB	CT	PR	No	3	5.6	20	40%	2018/3/15	9.0	Alive
P16	65	Female	No	IVB	CT	SD	No	7	2.4	0	10%	2015/8/9	40.7	Alive
P18	39	Female	No	IVB	CT	SD	No	0	1.6	80	10%	2018/10/31	1.9	Alive
P10	40	Male	Smoker, 15	IVB	Not treated	–	No	2	0	5	5%	2018/3/29	7.1	Alive

– Data not available

*Adj CT* adjuvant chemotherapy,*CNV* copy number variation, *CT* chemotherapy, *Neo CT* neoadjuvant chemotherapy, *OS* overall survival, *PR* partial response, *RT* radiotherapy, *S* surgery, *SD* stable disease, *TMB* tumor mutation burden, *TPS* tumor positive score

^a^Patients P28 and P29 did not have adequate tissue samples for genomic profiling

### Genetic alterations detected in the cohort

**Fig. 1:**
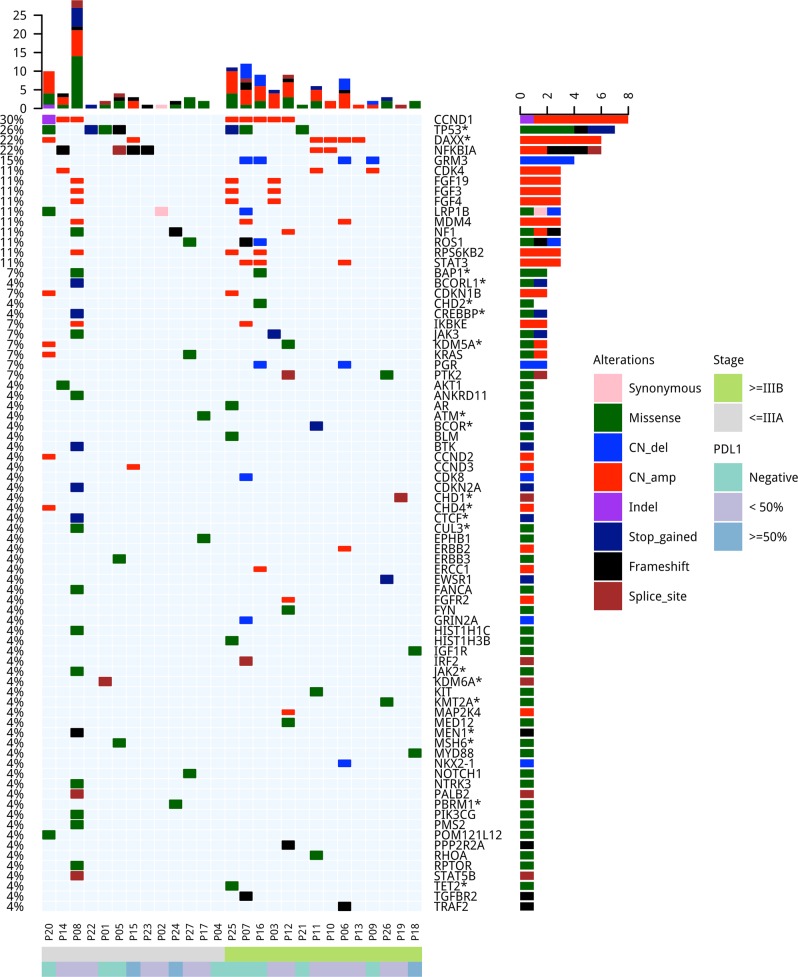
Mutation spectrum of the 27 Chinese pulmonary lymphoepithelioma-like carcinoma patients. The patients were grouped as either early-stage or advanced-stage as indicated by the bar located at the bottom of the oncoprint. The patient number and their corresponding PD-L1 expression were also indicated at the bottom of the oncoprint. Each column represents a patient and each row represents a gene. Genes related to epigenetic regulation were indicated with asterisks. Numbers on the left represent the percentage of patients with mutations in a specific gene. Top plot represents the overall number of mutations a patient carried. Different colors denote different types of mutation

Capture-based targeted next-generation sequencing was performed on archived tissue samples from 27 patients with adequate remaining samples to elucidate the comprehensive mutational profile of pulmonary lymphoepithelioma-like carcinoma tumors using a panel of 520 cancer-related genes, spanning 1.64 Mb of the human genome. Collectively, 184 genomic alterations in 109 genes were detected from 26 patients, including 107 single nucleotide variations, 12 insertions or deletions, and 65 copy number amplifications (Table S2). A patient (P04) had no mutation detected from this panel. Of the classic non-small-cell lung cancer oncogenic driver mutations, only two were detected in two patients, a *KRAS* G12D in a stage IIIA patient (P27, Fig. 1) and an *ERBB2* gene amplification in a stage IVA patient (P06, Fig. 1). No other actionable mutations were detected in the cohort. Most frequently mutated genes included *CCND1*, *TP53*, *DAXX*, and *NFκB1A*, occurring in 30% (8/27), 26% (7/27), 22% (6/27), and 22% (6/27) of the cohort, respectively. Interestingly, half of the cohort (51%, 14/27) had at least one copy number variation detected, with a majority detected among patients with advanced-stage disease (72%, 47/65 copy number variations in 10 advanced-stage patients vs. 28%, 18/65 in 4 early-stage patients; *P* = 0.057; Fig. 1, Tables 2 and 3). The copy number variations detected in the cohort included 7 *CCND1*, 5 *DAXX*, 4 *GRM3*, 3 *FGF19*, 3 *FGF3*, 3 *FGF4*, 3 *MDM4*, 3 *STAT3*, and 3 *RPS6KB2*. However, gene amplifications in *FGF19*, *FGF3*, *FGF4*, *MDM4*, *STAT3*, and *RPS6KB2* were found to be concurrent with gene amplifications of either *CCND1* or *DAXX* (*P* = 0.012, Fig. 1). In addition, *STAT3* amplifications were only detected among advanced-stage patients, with two of the *STAT3* concurrent with *CCND1* gene amplifications (P7 and P16, Fig. 1) and one concurrent with *DAXX* among other concurrent gene alterations (P6, Fig. 1). Furthermore, our data revealed that 78% (21/27) of the patients had mutations in epigenetic regulators. Of the 184 mutations identified, 51 mutations (28%, 51/184), occurred in 29 genes related to epigenetic regulation. The mutation frequency in epigenetics-related genes was similar between the early-stage and advanced-stage patients (*P* = 1). The epigenetics-related genes were identified using the EpiFactors database [35] and were labeled with asterisks in Fig. 1. Table S3 lists the 92 epigenetics-related genes included in the OncoScreen Plus panel based on the EpiFactors database.

### Tumor mutation burden in pulmonary lymphoepithelioma-like carcinoma patients

Next, we estimated tumor mutation burden for all the patients who underwent NGS-based molecular profiling. Tumor mutation burden was calculated as a ratio of the total nonsynonymous mutations per total size of the panel, excluding copy number variations and rearrangements as described in the “Methods” section. The median tumor mutation burden of the cohort was 1.6 mutations/Mb, ranging from 0 to 27.8 mutations/Mb. Three advanced-stage patients with only copy number variations had zero tumor mutation burden (Fig. 1 and Table 3). Tumor mutation burden was comparable between the early-stage and advanced-stage patients in the cohort (*P* = 0.69), with a median tumor mutation burden of 1.6 mutations/Mb and 2.0 mutations/Mb, respectively. The tumor mutation burden of patients in our cohort is significantly lower than that of lung adenocarcinoma patients included in The Cancer Genome Atlas (TCGA) dataset [36] (*P* < 0.01).

### Comparison with TCGA

**Fig. 2:**
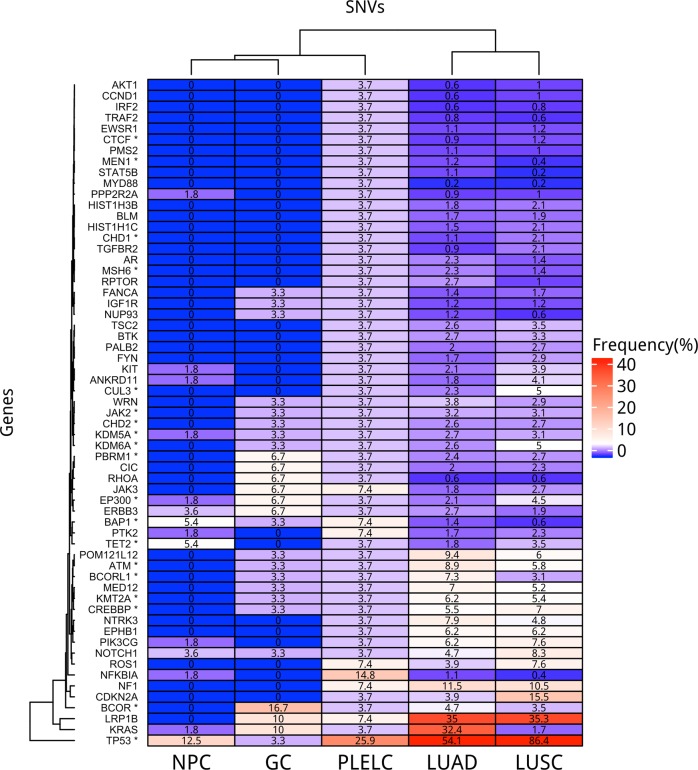
Pulmonary lymphoepithelioma-like carcinoma tumors have distinct mutational landscape. Heat map comparing mutation frequencies of pulmonary lymphoepithelioma-like carcinoma (PLELC) tumors in our cohort to lung adenocarcinoma (LUAD), lung squamous cell carcinoma (LUSC), nasopharyngeal carcinoma (EBV+ NPC), and gastric carcinoma (EBV+ GC) from The Cancer Genome Atlas dataset. Only single nucleotide variations (SNV) detected from genes listed were compared. Genes participating in epigenetic regulation were indicated with asterisks

**Fig. 3:**
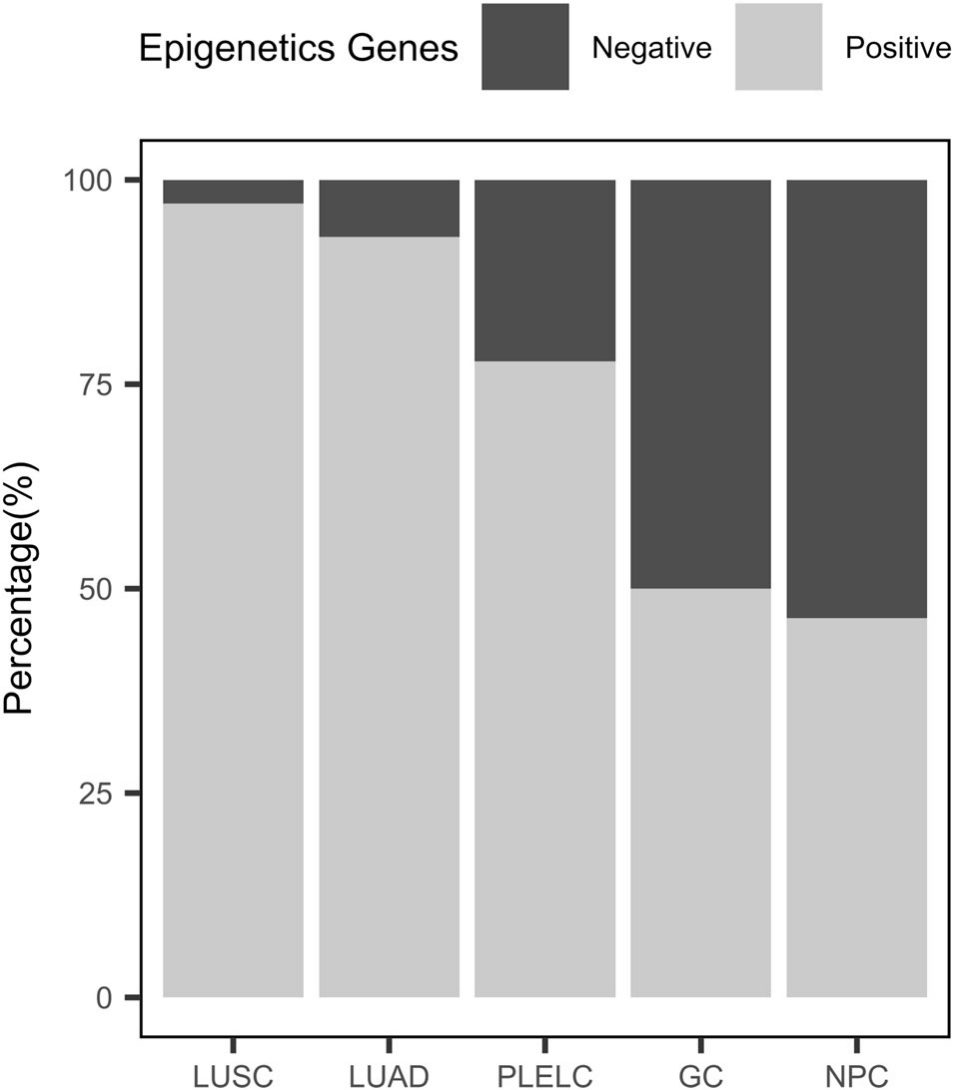
Pulmonary lymphoepithelioma-like carcinoma (PLELC) tumors have significantly different frequencies of mutations in genes related to epigenetic regulation as compared to lung adenocarcinoma (LUAD), lung squamous cell carcinoma (LUSC), EBV+ nasopharyngeal carcinoma (NPC), and EBV+ gastric carcinoma (GC) from The Cancer Genome Atlas dataset. The *x*-axis denotes tumor type. The *y*-axis denotes the frequency of single nucleotide variations detected in 92 epigenetic-related genes included in the panel used for genomic profiling

Next, we compared mutation profiles of pulmonary lymphoepithelioma-like carcinoma tumors from our cohort to mutation landscape of non-small cell lung cancer patients, as well as Epstein-Barr virus-positive (EBV+) nasopharyngeal carcinoma and EBV+ gastric cancer obtained from TCGA. The dataset for non-small-cell lung cancer has 1144 samples, comprised of 660 lung adenocarcinoma (LUAD) [36] and 484 squamous cell lung carcinoma samples (LUSC); [37] while the dataset for the EBV+ nasopharyngeal carcinoma (NPC) [38] and EBV+ gastric cancer (GC) [39] included 56 and 30 samples, respectively. Since the dataset from TCGA were generated through whole exome sequencing, analyses were only limited to the comparison of single nucleotide variants and excluded copy number variations and gene rearrangements. Figure 2 illustrates the mutation frequencies of lung adenocarcinoma, lung squamous cell carcinoma, EBV+ nasopharyngeal carcinoma, EBV+ gastric cancer dataset and our cohort. For the comparison, the genes with mutations detected from our cohort were extracted. With frequent mutations in epigenetic regulators observed in our cohort, we first examined whether this is a pulmonary lymphoepithelioma-like carcinoma-specific event or it is common among other EBV+ cancers. Comparing to EBV+ nasopharyngeal carcinoma (77.8 vs. 46.4%; *P* = 0.009) and EBV+ gastric cancer (77.8 vs. 50%; *P* = 0.053), pulmonary lymphoepithelioma-like carcinoma tumors had significantly more mutations in epigenetic-related genes (Fig. 3). In contrast, our analysis revealed significantly fewer mutations in epigenetics-related genes in pulmonary lymphoepithelioma-like carcinoma tumors as compared with other non-small cell lung cancer tumors (77.8% vs. LUAD = 93%; *P* = 0.012; LUSC = 97.1%; *P* < 0.001; Fig. 3).

In addition to differences in epigenetic regulator mutation profile, comparing to non-small cell lung cancer, pulmonary lymphoepithelioma-like carcinoma tumors had very limited mutations in classic lung cancer drivers. In our cohort, we only identified two driver mutations: one *KRAS* G12D and one *ERBB2* amplification. Furthermore, our analysis revealed that pulmonary lymphoepithelioma-like carcinoma had a significantly lower mutation frequencies in *TP53* (54.1 vs. 25.9%, *P* = 0.005), *KRAS* (32.4 vs. 3.7%, *P* < 0.001), and *LRP1B* (35.0 vs. 7.4%, *P* = 0.003) than lung adenocarcinoma. It also had a significant lower mutation frequencies in *TP53*, (86.4 vs. 25.9%, *P* < 0.001) and *LRP1B* (35.3 vs. 7.4%, *P* = 0.002) than lung squamous cell carcinoma. Pulmonary lymphoepithelioma-like carcinoma and EBV+ nasopharyngeal carcinoma had a comparable *TP53* mutation frequency (25.9 vs. 12.5%, *P* = 0.21) but it had a significantly higher *TP53* mutation frequency than EBV+ gastric tumors (25.9 vs. 3.3%, *P* = 0.021). Interestingly, mutations in *NFκB1A* were found to be significantly higher in pulmonary lymphoepithelioma-like carcinoma than all tumor types analyzed (PLELC = 14.8%; LUAD = 1.1%, *P* < 0.001; LUSC = 0.8%, *P* < 0.001; EBV+ NPC = 1.8%, *P* = 0.033; EBV+ GC = 0%, *P* < 0.001). Collectively, we elucidated a distinct genomic landscape associated with pulmonary lymphoepithelioma-like carcinoma with no classic non-small-cell lung cancer driver mutation but an enrichment of mutations in epigenetic regulators.

### Analysis of PD-L1 expression

We also assessed the PD-L1 expression status of all the patients in our cohort using the Dako 22C3 immunostaining assay. Analysis revealed that 66% (20/29) of the tumors had significant PD-L1 membrane staining, including 14% (4/29) with high PD-L1 expression (≥50%) and 55% (16/29) with low PD-L1 expression (5–49%). The remaining 31% (9/29) of the patients were negative for PD-L1 immunostaining (Table 1). PD-L1 levels between the early-stage and advanced-stage patients were also comparable (*P* = 1). No significant association was found between the PD-L1 expression and tumor mutation burden (*P* = 0.57, Table 3). Further correlation analysis revealed significantly lower PD-L1 expression among *TP53*-mutant patients (*P* < 0.001, Fig. S2). In our cohort, seven patients were *TP53*-mutant and six of them had no PD-L1 expression. In the *TP53*-wild-type group, only three patients (3/20) had no PD-L1 expression.

### Immunotherapy for pulmonary lymphoepithelioma-like carcinoma

Three patients from our cohort were administered with PD-1 inhibitor either alone or in combination with chemotherapy after failures from at least one line of treatment. These information are summarized in Table 2 (P08) and Table 3 (P28 and P29).

Patient P08 was a 56-year-old female, never smoker, who was diagnosed with stage IIB pulmonary lymphoepithelioma-like carcinoma in October 2017 and had relapse after a year. She had PD-L1 expression of 30% and tumor mutation burden of 27.8 mutations/Mb. Subsequently, she started on nivolumab in combination with gemcitabine. After 4 weeks of treatment, computed tomography (CT) scans revealed stable disease. She was lost to follow-up after her last radiographic evaluation.

Patient P28 was a 49-year-old female, never smoker, diagnosed with stage IIIB pulmonary lymphoepithelioma-like carcinoma on April 12, 2017. PD-L1 staining revealed tumor proportion score of 60%. After failure from first-line chemotherapy, she was administered with nivolumab monotherapy in October 2018 and achieved stable disease 4 weeks after the initiation of the treatment. CT scans in December 2018 revealed enlargement of the primary lung lesions. Subsequently, she was switched to nivolumab in combination with anlotinib and gemcitabine and achieved stable disease (Fig. S3A–C). She still remains on this regimen as of April 19, 2019.

Patient P29 was a 48-year-old male, diagnosed with stage IVA disease in February 28, 2017, with PD-L1 expression of 15%. After failure from two lines of chemotherapy, he was enrolled in an investigational trial for camrelizumab (SHR-1210), a monoclonal antibody against PD-1, combined with apatinib in September 2018, and achieved stable disease (Fig. S4A–C). He still remains on the treatment as of April 19, 2019.

## Discussion

To the best of our knowledge, our study is the first to elucidate the comprehensive mutational profile of pulmonary lymphoepithelioma-like carcinoma tumors in order to shed light on the molecular mechanism of its tumorigenesis. Numerous studies have examined the involvement of classic lung cancer oncogenic pathways in the development of pulmonary lymphoepithelioma-like carcinoma, including *EGFR* [4, 6, 7, 15, 23, 24], *KRAS*, *BRAF*, *ALK*, and *ROS1* [23] and discovered that these pathways are not major oncogenic drivers of pulmonary lymphoepithelioma-like carcinoma. Despite the *EGFR* mutation rate of 12.1% (8/66) reported by Chang et al., only one patient was detected with a sensitizing *EGFR* mutation—an *EGFR* exon 19 deletion; all the other 11 *EGFR* mutations detected in 7 pulmonary lymphoepithelioma-like carcinoma patients located in exons 18–21 are uncommon mutations with no evidence of therapeutic response to EGFR inhibitors [4, 23, 24]. These observations strongly suggest that the EGFR pathway is not involved in the tumor development of pulmonary lymphoepithelioma-like carcinoma [4, 23, 24]. In addition to oncogenic drivers, TP53 mutations E298X, R273C, and G279R were also detected in three patients, resulting in a TP53 mutation rate of 6.5% (3/46) [4]. In our study, instead of interrogating only the classic lung cancer oncogenic drivers, we used ultra-deep capture-based targeted sequencing with a panel consisting of 520 cancer-related genes to obtain a more comprehensive mutation profile of pulmonary lymphoepithelioma-like carcinoma. Our analysis revealed the detection of two mutations in oncogenic drivers, *KRAS* G12D and *ERBB2* amplifications from two patients. No other mutation in classic non-small cell lung cancer oncogenic driver genes was identified from our cohort. Consistent with previous reports, our findings suggest that classic oncogenic drivers of lung cancer are not primary contributors to the tumorigenesis of pulmonary lymphoepithelioma-like carcinoma. Instead, we revealed an enrichment of mutations in epigenetic regulators, occurring in 78% (21/27) of the patients, indicating that chromatin remodeling and modification might be involved in the development of pulmonary lymphoepithelioma-like carcinoma tumors. Epigenetic mechanisms including DNA methylation, histone post-translation modification, and chromatin remodeling can regulate gene expression [40]. In addition to genetic factors, alterations in epigenetic regulation contribute significantly to the initiation and progression of cancer [40, 41]. Epigenetic pathways are also implicated in the observed intratumor heterogeneity [40] and alterations in the tumor microenvironment [42].

Morphologically, lymphoepithelioma is characterized by the presence of clusters of epithelial tumor cells with round, oval or elongated nuclei, surrounded by lymphoid cells including lymphocytes and granulocytes [11]. Consistent with the findings by Yeh et al. [15], we have found varying degrees of lymphocytic infiltration in the tumors of our cohort. Extensive lymphoid infiltration can be observed in the specimen of most patients and made the pathological diagnosis convenient. However, in five patients with very few lymphoid cells surrounding the tumor cells, EBER in situ hybridization was a key determinant in confirming the pathological diagnosis.

Pulmonary lymphoepithelioma-like carcinoma has been classified as a rare type of non-small cell lung cancer with histological resemblance to EBV- positive nasopharyngeal carcinoma [3, 11, 13]. Hence, we have compared the mutation profile of the tumors from our cohort with non-small cell lung cancer (adenocarcinoma and squamous cell carcinoma) as well as two EBV-associated malignancies (EBV+ nasopharyngeal carcinoma and EBV+ gastric cancer) from TCGA dataset. Our analysis further confirmed that pulmonary lymphoepithelioma-like carcinoma had a distinct mutation profile. Pulmonary lymphoepithelioma-like carcinoma tumors harbored significantly more mutations in epigenetic regulators than EBV+ nasopharyngeal carcinoma (77.8 vs. 46.4%; *P* = 0.009) and have a trend of having more mutations in epigenetic regulators in EBV+ gastric cancer tumors (77.8 vs. 50%; *P* = 0.053). Furthermore, very limited mutations were shared by pulmonary lymphoepithelioma-like carcinoma and EBV+ nasopharyngeal carcinoma as well as pulmonary lymphoepithelioma-like carcinoma and EBV+ gastric cancer. However, since TCGA dataset were derived from whole exome sequencing, only single nucleotide variations were analyzed and compared. It should be noted that approximately half of the patients in our cohort had at least one copy number variation. Of which, *CCND1* amplifications were the most predominant, with a mutation rate of 30% (8/27). Copy number variations are associated with various human cancers [43, 44]. In particular, gene amplification in *CCDN1* is considered as one of the key drivers in lung carcinogenesis by regulating cell cycle progression [45].

Due to the rareness of the advanced-stage pulmonary lymphoepithelioma-like carcinoma, currently, there is no consensus on the chemotherapy regimen and radiation dose for advanced-stage disease. Our previous study has revealed that pulmonary lymphoepithelioma-like carcinoma had a better prognosis compared with other types of non-small-cell lung cancer and was sensitive to radiotherapy and chemotherapy but not to targeted EGFR-TKI therapy [9]. Moreover, advanced-stage pulmonary lymphoepithelioma-like carcinoma patients treated with platinum-based chemotherapy in combination with either paclitaxel (TP, 9/33) or gemcitabine (GP, 16/33) had significantly longer progression-free survival than those treated with pemetrexed plus platinum-based chemotherapy (PP, 8/33) (*P* = 0.001), strongly suggesting that TP and GP regimens as first-line therapy provide more durable clinical benefit to pulmonary lymphoepithelioma-like carcinoma patients than PP regimen [9]. In addition to chemotherapy and radiotherapy, scientists are actively exploring other treatment regimens, including but not limited to targeted therapy and immunotherapy. The introduction of immune checkpoint inhibitors in clinical practice has revolutionized the treatment of cancer patients. Numerous studies have consistently demonstrated long-term tumor responses from PD-1/PD-L1 inhibitors as compared with conventional chemotherapy in patients with PD-L1-positive tumors [46] or in some cancer types regardless of PD-L1 status [47]. PD-L1 positivity, defined as the presence of moderate to strong membrane staining in at least 5% of the tumor cells, is considered to be a predictive biomarker for checkpoint immunotherapy response in various cancer types [33, 34]. Virus-associated malignancies including EBV+ gastric cancer, EBV+ nasopharyngeal carcinoma, Merkel-cell carcinoma and hepatocellular carcinoma has been demonstrated to be responsive to checkpoint immunotherapy [48–50]. A study that assessed 66 Taiwanese pulmonary lymphoepithelioma-like carcinoma patients reported that 75.8% (50/66) were PD-L1 positive [23]. Consistently, PD-L1 was also expressed on the surface of the tumors of 69% (20/29) patients in our cohort, including 14% (4/29) with high PD-L1 expression (≥50%) and 55% (16/29) with moderate PD-L1 expression level (5–49%). Despite the low tumor mutation burden in pulmonary lymphoepithelioma-like carcinoma tumors, the PD-L1 positivity of a majority of the tumors raises the potential of utilizing checkpoint immunotherapy as a treatment regimen that could benefit these patients. The administration of immunotherapy in the three patients with PD-L1 tumor proportion scores of 15, 30, and 60%, all achieving stable disease, indicates that checkpoint immunotherapy can be beneficial to pulmonary lymphoepithelioma-like carcinoma patients and can be a treatment option for this subset of patients.

Consistent with our findings, investigations on patients with lymphoepithelioma-like carcinoma of the thymus have also revealed the absence of actionable genes as well as the detection of various degrees of membranous PD-L1 staining in their cohort (total: 71.4%, 15/21; high expression (>50% of tumor cells): 48%; low expression (<50% of tumor cells): 25%) [51, 52]. Moreover, they have observed that the patients with PD-L1 expression contained abundant lymphocytes in the stroma, despite the lack of EBV positivity [52]. In contrast to their report, we did not find a correlation between the PD-L1 expression and extent of lymphocytic infiltration in our cohort (Tables 2 and 3). With the disease control achieved by the administration of immunotherapy in the three patients in our cohort and the similarities in the tumor biology between lymphoepithelioma-like carcinoma of the thymus and lung indicate that checkpoint immunotherapy should be included in the clinical management of patients with these rare lymphoepithelioma-like carcinoma tumors.

Taken together, our findings reveal that the genomic landscape of pulmonary lymphoepithelioma-like carcinoma is distinctive to non-small-cell lung cancer, EBV positive nasopharyngeal carcinoma and EBV positive gastric cancer, with the detection of only two classic lung cancer driver mutations but an enrichment of mutations in epigenetic regulators. The observation of high PD-L1 expression and lack of canonical druggable driver mutation raises the potential of checkpoint immunotherapy for this rare tumor type. A clinical trial evaluating the treatment response of pulmonary lymphoepithelioma-like carcinoma patients to checkpoint immunotherapy is currently being registered by our group. However, due to its rarity, a larger multicenter study is still needed to enroll more patients and obtain clinically relevant results.

## Supplementary information

Supplementary Tables

Supplementary Figures

Supplemental Figure and Table Legends
