# Supplementary material for: A multicenter analysis of genomic profiles and PD-L1 expression of primary lymphoepithelioma-like carcinoma of the lung
Source: Mod Pathol. 2019 Oct 28;33(4):626–38. doi: 10.1038/s41379-019-0391-9 (PMC7113185; doi:10.1038/s41379-019-0391-9)
Supplement: Supplementary file 1 — Supplementary Tables [file 41379_2019_391_MOESM1_ESM.docx]

Table S1. List of the 520 cancer-related genes included in the OncoScreen Plus panel

| ***ABL1*** | ***BCL2L1*** | ***CDKN2B*** | *EGFL7* | *FAS* | ***HIST1H3B*** | ***IRS1*** | ***MDM2*** | *NOTCH4* | *PIK3R3* | ***RAD54L*** | ***SMAD4*** | *TMEM127* |
| --- | --- | --- | --- | --- | --- | --- | --- | --- | --- | --- | --- | --- |
| *ABL2* | *BCL2L11* | ***CDKN2C*** | ***EGFR*** | ***FAT1*** | ***HIST1H3C*** | ***IRS2*** | ***MDM4*** | ***NPM1*** | *PIM1* | ***RAF1*** | ***SMARCA4*** | *TMPR552* |
| *ACVR1* | *BCL2L2* | ***CEBPA*** | *EIF1AX* | ***FAT3*** | ***HIST1H3D*** | ***JAK1*** | ***MED12*** | ***NRAS*** | ***PLCG2*** | *RANBP2* | ***SMARCB1*** | ***TNFAIP3*** |
| *ACVR1B* | ***BCL6*** | *CENPA* | *EIF4A2* | ***FBXW7*** | ***HIST1H3E*** | ***JAK2*** | ***MEF2B*** | *NR4A3* | *PLK2* | ***RARA*** | *SMARCD1* | ***TNFRSF14*** |
| *ADGRA2* | ***BCOR*** | ***CHD1*** | *EIF4E* | *FCGR2B* | *HIST1H3F* | ***JAK3*** | ***MEN1*** | ***NRG1*** | *PMAIP1* | *RASA1* | ***SMO*** | ***TNFSF11*** |
| ***AKT1*** | *BCORL1* | *CHD2* | *ELOC* | *FGF10* | ***HIST1H3G*** | ***JUN*** | ***MET*** | ***NSD1*** | ***PMS1*** | ***RB1*** | *SNCAIP* | ***TOP1*** |
| ***AKT2*** | *BCR* | *CHD4* | ***EMSY*** | *FGF12* | ***HIST1H3H*** | *KAT5A* | *MGA* | ***NTHL1*** | ***PMS2*** | ***RBM10*** | ***SOCS1*** | *TOP2A* |
| ***AKT3*** | ***BLM*** | ***CHEK1*** | ***EP300*** | *FGF23* | ***HIST1H3I*** | ***KDM5A*** | ***MITF*** | ***NTRK1*** | *PNRC1* | *RECQL4* | ***SOX2*** | ***TP53*** |
| ***ALK*** | *BMPR1A* | ***CHEK2*** | *EPCAM* | *FGF6* | ***HIST1H3J*** | ***KDM5C*** | ***MLH1*** | ***NTRK2*** | ***POLD1*** | *REL* | ***SOX9*** | *TRAF2* |
| *ALOX12B* | ***BRAF*** | *CHUK* | *EPHA2* | *FGF7* | *HIST2H3C* | ***KDM6A*** | ***MLH3*** | ***NTRK3*** | ***POLE*** | ***RET*** | *SOX10* | *TRAF7* |
| ***AMER1*** | ***BRCA1*** | ***CIC*** | *EPHA3* | *FGFR14* | ***HIST2H3D*** | ***KDR*** | ***MPL*** | ***NUP93*** | ***POM121L12*** | *RFWD2* | *SOX17* | *TRRAP* |
| *ANKRD11* | ***BRCA2*** | *CRBN* | *EPHA5* | *FGFR1* | ***HIST3H3*** | ***KEAP1*** | ***MRE11A*** | *PAK1* | *PPM1D* | *RHEB* | ***SPEN*** | ***TSC1*** |
| ***APC*** | ***BRD4*** | ***CREBBP*** | ***EPHA7*** | *FGFR2* | ***HLA-A*** | ***KEL*** | ***MSH2*** | *PAK3* | ***PPP2R1A*** | *RHOA* | ***SPOP*** | ***TSC2*** |
| *APCDD1* | ***BRIP1*** | ***CRKL*** | ***EPHB1*** | *FGFR3* | ***HNF1A*** | ***KIT*** | ***MSH3*** | *PAK7* | ***PPP2R2A*** | ***RICTOR*** | ***SPTA1*** | ***TSHR*** |
| ***AR*** | *BTG1* | ***CRLF2*** | ***ERBB2*** | *FOXA1* | ***HNF1B*** | *KLF4* | ***MSH6*** | ***PALB2*** | *PPP6C* | *RIT1* | ***SRC*** | ***U2AF1*** |
| ***ARAF*** | ***BTK*** | ***CSF1R*** | ***ERBB3*** | ***FOXL2*** | *HOXB13* | *KLHL6* | *MST1* | ***PARK2*** | ***PRDM1*** | ***RNF43*** | ***SRSF2*** | ***VEGFA*** |
| *ARFRP1* | *CALR* | *CSF3R* | *ERBB4* | *FRS2* | ***HRAS*** | ***KMT2A*** | *MST1R* | *PARP1* | *PREX2* | ***ROS1*** | ***STAG2*** | *VEGFB* |
| ***ARID1A*** | ***CARD11*** | ***CTCF*** | ***ERBB5*** | *FYN* | *HSD3B1* | ***KMT2C*** | ***MTOR*** | *PARP2* | ***PRKAR1A*** | *RPA1* | ***STAT3*** | *VEGFC* |
| ***ARID1B*** | *CASP8* | *CTLA4* | ***ERCC1*** | *GABRA6* | ***HSP90AA1*** | ***KMT2D*** | ***MUTYH*** | *PARP3* | *PRKC1* | *RPS6KA4* | *STAT4* | ***VHL*** |
| ***ARID2*** | ***CBFB*** | *CTNNA1* | *ERCC2* | *GATA4* | *ICOSLG* | ***KRAS*** | ***MYC*** | *PARP4* | ***PRKDC*** | *RPS6KB2* | *STAT5A* | *VTCN1* |
| *ARID5B* | ***CBL*** | ***CTNNB1*** | *ERCC3* | *GATA6* | *ID3* | ***LATS1*** | ***MYCL*** | ***PAX5*** | *PRSS8* | ***RPTOR*** | ***STAT5B*** | *WISP3* |
| ***ASXL1*** | ***CCND1*** | ***CUL3*** | *ERCC4* | *GID4* | ***IDH1*** | *LATS2* | ***MYCN*** | ***PBRM1*** | ***PTCH1*** | ***RUNX1*** | ***STK11*** | ***WRN*** |
| *ASXL2* | ***CCND2*** | *CUL4A* | *ERCC5* | *GNA13* | ***IDH2*** | ***LMO1*** | ***MYD88*** | *PDCD1* | ***PTEN*** | *RUNX1T1* | *STK40* | ***WT1*** |
| *ATF1* | ***CCND3*** | *CUL4B* | ***ERG*** | *GPS2* | *IFNGR1* | ***LRP1B*** | *MYOD1* | *PDCD1LG2* | *PTK2* | *RYBP* | ***SUFU*** | *XIAP* |
| ***ATM*** | ***CCNE1*** | *CXCR4* | ***ERRFI1*** | ***GREM1*** | *IGF1* | *LYN* | ***NBN*** | ***PDFRA*** | ***PTPN11*** | ***SDHA*** | *SUZ12* | ***XPO1*** |
| ***ATR*** | ***CD274*** | *CYCLD* | ***ESR2*** | ***GRM3*** | ***IGF1R*** | *LZTR1* | *NCOA3* | ***PDGFRB*** | ***PTPRD*** | *SDHAF2* | ***SYK*** | ***XRCC2*** |
| ***ATRX*** | *CD276* | *CYP17A1* | *EWSR1* | ***GSK3B*** | ***IGF2*** | *MAG12* | *NCOR1* | *PDK1* | *PTPRS* | ***SDHB*** | *TACC3* | *XRCC3* |
| ***AURKA*** | ***CD79A*** | ***DAXX*** | ***EZH2*** | ***GSTM1*** | ***IKBKE*** | ***MALT1*** | *NEB* | *PGR* | *PTPRT* | ***SDHC*** | *TAF1* | *YAP1* |
| ***AURKB*** | ***CD79B*** | *DCUN1D1* | ***FAM175A*** | ***GSTT1*** | ***IKZF1*** | ***MAP2K1*** | *NEGR1* | *PHOX2B* | *QK1* | ***SDHD*** | ***TBX3*** | *YES1* |
| ***AXIN1*** | ***CDC73*** | ***DDR2*** | ***FAM46C*** | ***H3F3A*** | *IL10* | ***MAP2K2*** | ***NF1*** | ***PIK3CA*** | *RAB35* | ***SETD2*** | *TCF3* | *ZBTB2* |
| *AXIN2* | ***CDH1*** | ***DICER1*** | ***FANCA*** | *H3F3B* | ***IL7R*** | ***MAP2K4*** | ***NF2*** | ***PIK3CB*** | ***RAC1*** | ***SF3B1*** | *TCF7L2* | *ZFHX3* |
| ***AXL*** | ***CDK12*** | *DIS3* | ***FANCC*** | *HDAC1* | ***INHA*** | ***MAP3K1*** | ***NFE2L2*** | *PIK3C2B* | *RAD21* | *SH2B3* | ***TERC*** | *ZNF217* |
| *B2M* | ***CDK4*** | *DNAJB1* | ***FANCD2*** | *HDAC2* | ***INHBA*** | *MAP3K13* | ***NFKB1A*** | *PIK3C2G* | ***RAD50*** | *SH2D1A* | ***TERT*** | *ZNF703* |
| *BACH1* | ***CDK6*** | *DNMT1* | ***FANCE*** | *HDAC4* | *INPP4A* | *MAP3K14* | ***NKX2-1*** | *PIK3C3* | ***RAD51*** | *SHQ1* | *TET1* | *ZNRF3* |
| ***BAP1*** | ***CDK8*** | ***DNMT3A*** | ***FANCF*** | ***HGF*** | ***INPP4B*** | *MAP3K3* | *NKX3-1* | *PIK3CD* | ***RAD51B*** | *SLIT2* | ***TET2*** | *ZRSR2* |
| ***BARD1*** | ***CDKN1A*** | *DNMT3B* | ***FANCG*** | ***HIST1H1C*** | *INSR* | *MAPK1* | ***NOTCH1*** | ***PIK3CG*** | ***RAD51C*** | ***SLX4*** | *TGFBR1* |  |
| *BBC3* | ***CDKN1B*** | ***DOT1L*** | ***FANCI*** | ***HIST1H2BD*** | *IRF2* | *MAX* | ***NOTCH2*** | ***PIK3R1*** | ***RAD51D*** | ***SMAD2*** | ***TGFBR2*** |  |
| ***BCL2*** | ***CDKN1C*** | *E2F3* | ***FANCL*** | ***HIST1H3A*** | ***IRF4*** | ***MCL1*** | ***NOTCH3*** | ***PIK3R2*** | ***RAD52*** | ***SMAD3*** | *TIPARP* |  |
| *BCL10* | ***CDKN2A*** | *EED* | *FANCM* |  |  |  |  |  |  |  |  |  |

Note: Genes in bold denote the interrogation of whole exons. Genes in regular face denote the interrogation of critical exons, introns and promoter region. Genes in red indicate detection of fusion. Blue shading denotes the detection of single nucleotide variations and copy number variations. Green shading denotes detection of only single nucleotide variations. No shading means no other mutations are detected for the specified genes except fusions.

Table S2. Mutation types detected in the cohort

| Mutation type | Number of mutations |
| --- | --- |
| Missense | 48 |
| Nonsense | 10 |
| Splice site | 7 |
| Nonsynonymous variants detected in introns | 13 |
| Synonymous | 29 |
| Small insertion deletion (including disruptive indels) | 2 |
| Frameshift | 10 |
| Fusion | 0 |
| Copy number deletion | 12 |
| Copy number amplification | 53 |
| Total | 184 |

Table S3. List of the 92 epigenetic-related genes included in the OncoScreen Plus panel based on the EpiFactors database (ref.35)

| Genes | Function | Genes | Function | Genes | Function |
| --- | --- | --- | --- | --- | --- |
| ARID1A | Chromatin remodelling cofactor | DNMT3B | DNA mod. | NCOR1 | Histone mod. erase cofactor |
| ARID1B | Histone mod. write | DOT1L | Histone mod. write | NPM1 | Histone chaperone |
| ARID2 | Chromatin remodelling cofactor | EED | PcG protein | NSD1 | Histone mod. write |
| ASXL1 | Histone mod. erase; PcG protein | EP300 | Histone mod. write | PARP1 | Chromatin remodelling |
| ASXL2 | Histone mod. read | ERBB4 | Histone mod. cofactor | PARP2 | Chromatin remodelling cofactor |
| ATM | Histone mod. write | EZH2 | Histone mod. write; PcG protein | PARP3 | PcG protein |
| ATR | Histone mod. write | FAM175A | Scaffold protein | PBRM1 | Histone mod. read |
| ATRX | Chromatin remodelling | FOXA1 | Chromatin remodelling; TF | PRDM1 | Histone mod. write cofactor |
| AURKA | Histone mod. write | FOXO1 | TF | PRKDC | Histone mod. write |
| AURKB | Histone mod. write | FOXP1 | TF | RAD51 | Histone mod. erase |
| BAP1 | Histone mod. erase; PcG protein | HDAC1 | Histone mod. erase | RAD54L | Chromatin remodelling |
| BARD1 | Histone mod. write | HDAC2 | Histone mod. erase | RARA | Histone mod. write cofactor; TF |
| BCOR | PcG protein | HDAC4 | Histone mod. erase | RB1 | Chromatin remodelling; Histone mod. write |
| BCORL1 | Histone mod. erase cofactor | IKZF1 | Chromatin remodelling; TF | RPS6KA4 | Histone mod. write |
| BRCA1 | Histone mod. write cofactor; TF | JAK2 | Histone mod. write | RYBP | PcG protein |
| BRCA2 | Histone mod. write | KAT6A | Histone mod. write | SETD2 | Histone mod. write |
| BRD4 | Histone mod. read | KDM5A | Histone mod. erase | SF3B1 | RNA mod. |
| CDC73 | Histone mod. write cofactor | KDM5C | Histone mod. erase | SMARCA4 | Histone mod. read; TF |
| CHD1 | Chromatin remodelling | KDM6A | Histone mod. erase | SMARCB1 | Histone mod. read |
| CHD2 | Chromatin remodelling | KEAP1 | Chromatin remodelling | SMARCD1 | Chromatin remodelling |
| CHD4 | Chromatin remodelling | KMT2A | Histone mod. write | SPEN | Histone mod. erase cofactor; TF; TF |
| CHEK1 | Histone mod. write | KMT2C | Histone mod. write | SPOP | Histone mod. write |
| CHUK | Histone mod. write | KMT2D | Histone mod. write | SUZ12 | Histone mod. write cofactor; PcG protein; TF |
| CREBBP | Histone mod. write | MAX | Histone mod. write cofactor; TF | TAF1 | Histone mod. write |
| CTCF | Chromatin remodelling; TF | MDC1 | Histone mod. read | TET1 | DNA mod. |
| CUL3 | Histone mod. write | MEN1 | Histone mod. write cofactor | TET2 | DNA mod. |
| CUL4A | Histone mod. write | MGA | Histone mod. write cofactor; TF | TOP2A | Chromatin remodelling |
| CUL4B | Histone mod. write | MSH6 | Histone mod. read | TP53 | Histone mod. write cofactor; TF |
| DAXX | # | MST1 | Histone mod. | TRRAP | Histone mod. write cofactor |
| DNMT1 | DNA mod. | NBN | Chromatin remodelling | ZNF217 | Histone mod. erase cofactor; TF |
| DNMT3A | DNA mod. | NCOA3 | Histone mod. write |  |  |

Abbreviations: mod,. modification; PcG, Polycomb group; TF, transcription factor
