# Supplementary material for: A multicenter analysis of genomic profiles and PD-L1 expression of primary lymphoepithelioma-like carcinoma of the lung
Source: Mod Pathol. 2019 Oct 28;33(4):626–38. doi: 10.1038/s41379-019-0391-9 (PMC7113185; doi:10.1038/s41379-019-0391-9)
Supplement: Supplementary file 2 — Supplementary Figures [file 41379_2019_391_MOESM2_ESM.docx]

**
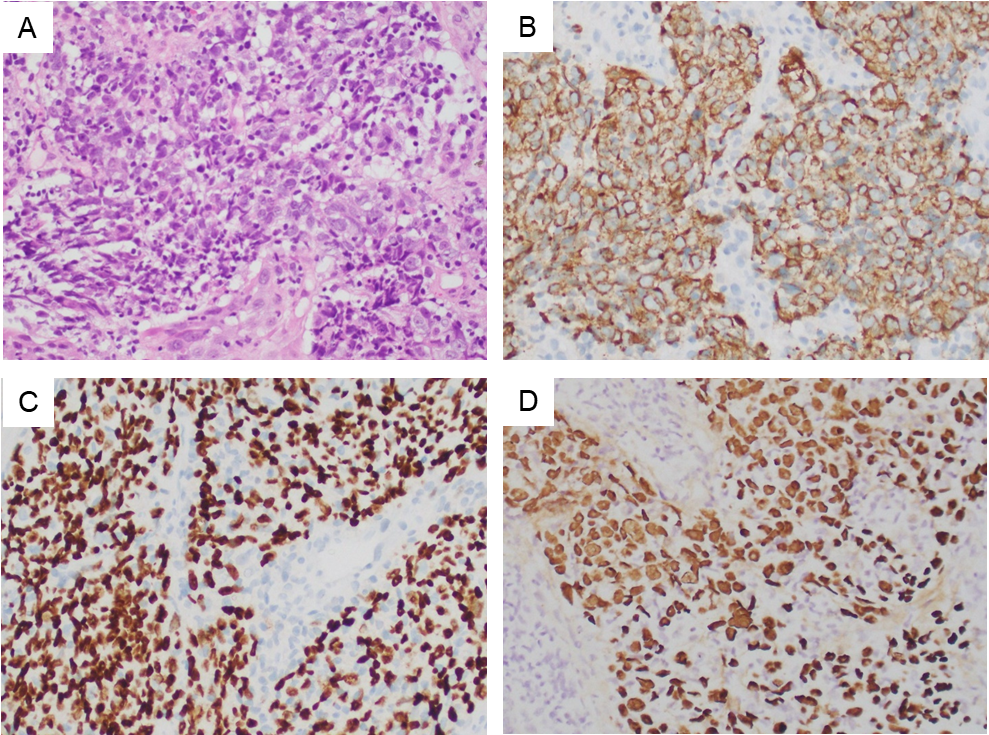
**

**Figure S1**. Histologic features of PLELC. A. Hematoxylin-eosin (HE) stain (magnification X400); B-C. Immunohistochemical staining of cytokeratin 5/6 (B) and P63 (magnification X200) (C); D. In situ hybridization of Epstein-Barr virus-encoded small non-polyadenylated RNA (EBER) (magnification X200).

**
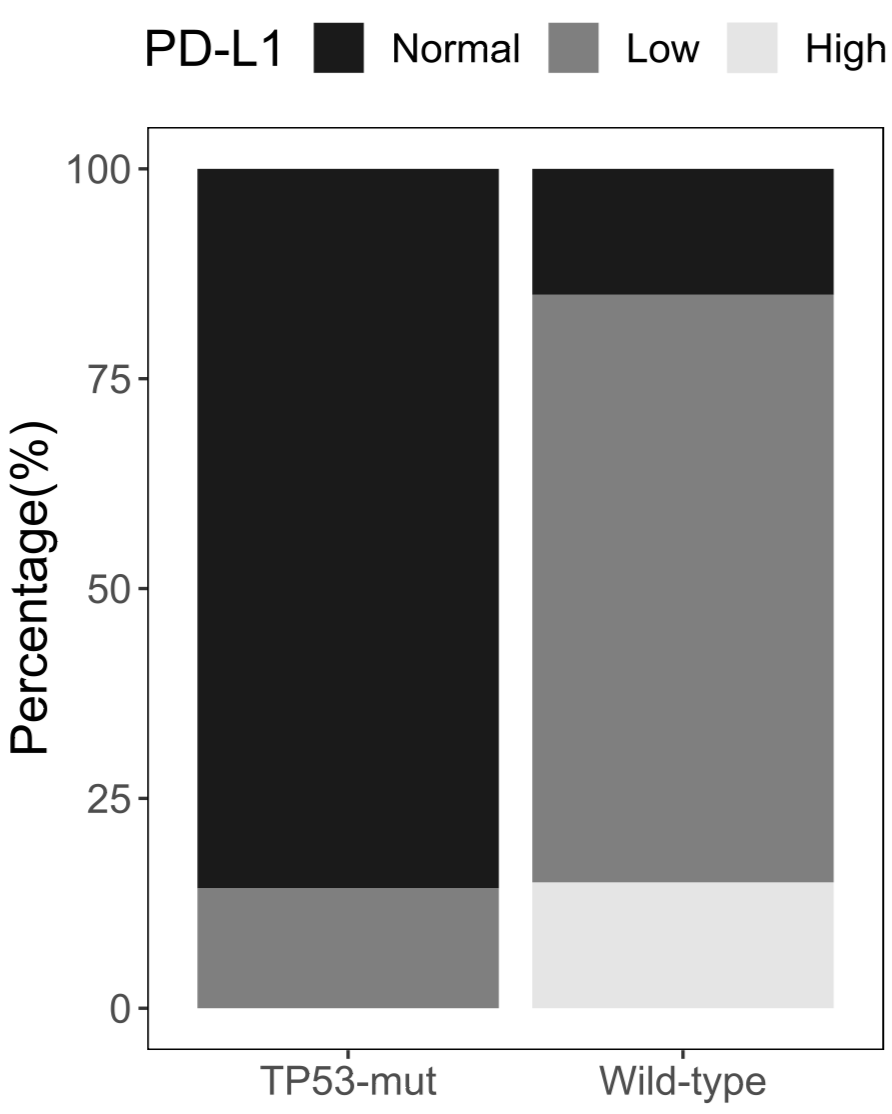
**

**Figure S2.** *TP53*-mutant patients were less likely to have PD-L1-positive tumor cells. The x-axis denotes *TP53* mutation status. The y-axis denotes the percentage of patients. Black shading represents negative PD-L1 expression; Medium dark gray shading represents low PD-L1 expression or TPS= 5-49% and Light gray shading represents high PD-L1 expression or TPS>50%.

**
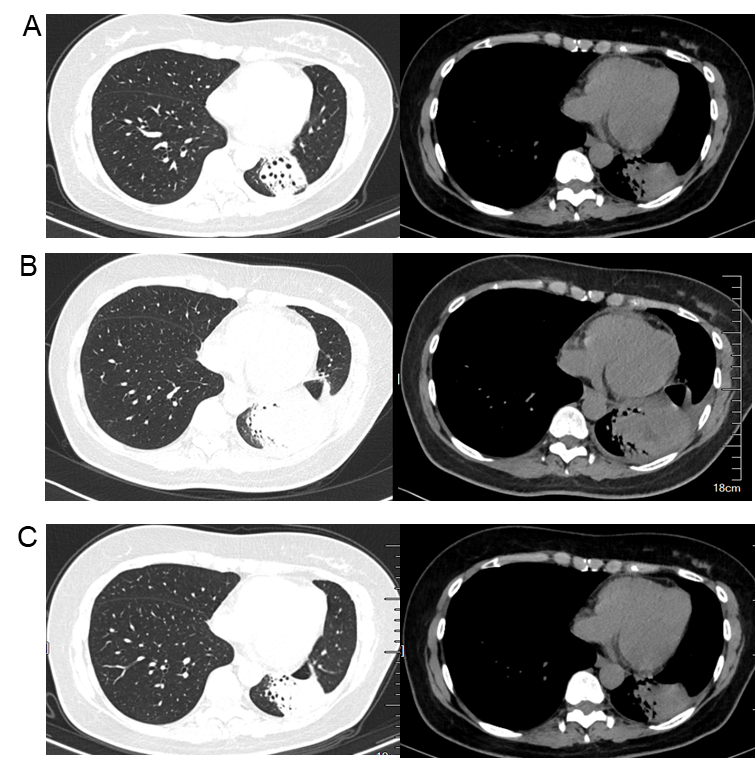
**

**Figure S3.** Computed tomography scans of Patient P28 showing the tumor responses to PD-1 inhibitor, nivolumab. A. Scans after chemotherapy regimen, with the disease evaluated as SD and prior to the switch to immunotherapy (Sep. 23, 2018). B. Scans after 6 cycles of nivolumab alone with tumor diameter of 5.7cm x 3.6cm. C. Scans after 4 cycles of nivolumab in combination with anlotinib and gemcitabine, with primary lung lesion in the lower left lobe reduced to 3.7cm x 3.0cm, indicating stable disease.

**
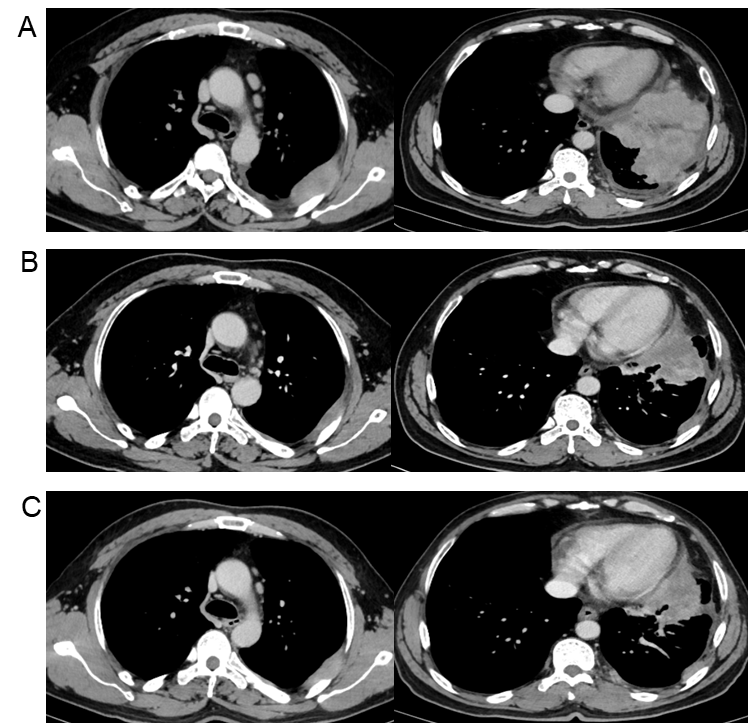
**

**Figure S4**. Computed tomography scans of Patient P29 showing the tumor responses to PD-1 inhibitor, SHR-1201. A. Scans demonstrating the large left lower lobe primary lesion, left hilar lymphadenopathy and metastatic spread to the left pleura (Aug. 29, 2018). B. Scans after 4 cycles of SHR-1201 with apatinib, with reduction of the primary lesion in the left lower lobe and left pleural metastasis. C. Scans after 6 cycles of SHR-1201 regimen with no observable enlargement in primary lung lesions in the left lower lobe, indicating stable disease.
